# Hsa_circ_0053063 inhibits breast cancer cell proliferation via hsa_circ_0053063/hsa-miR-330-3p/PDCD4 axis

**DOI:** 10.18632/aging.202707

**Published:** 2021-03-19

**Authors:** Changle Ji, Jiashu Hu, Xuehui Wang, Wenfang Zheng, Xiaochong Deng, Hongming Song, Yunhe Yu, Qifeng Luo, Kaiyao Hua, Xiqian Zhou, Lin Fang

**Affiliations:** 1 Department of Breast and Thyroid Surgery, Shanghai Tenth People's Hospital, Tongji University School of Medicine, Shanghai 200072, China; 2 Tongji University School of Medicine, Shanghai 200092, China; 3 Nanjing Medical University, Nanjing 211166, China; 4 Breast Disease Center, The Affiliated Hospital of Qingdao University, Qingdao 266000, Shandong, China

**Keywords:** breast cancer, hsa_circ_0053063, hsa-miR-330-3p, PDCD4, ceRNA, HADH

## Abstract

Breast cancer (BC) is one of the most common malignancies and its mortality is the highest among females. Circular RNAs (circRNAs), a novel group of non-coding RNAs, play an important regulatory role in angiogenesis and cancer progression. Hsa_circ_0053063 is a circRNA generated from several exons of HADHA. The potential role of hsa_circ_0053063 in BC remains unknown and needs to be explored. Hsa_circ_0053063 was mainly located in the cytoplasm and activated in BC tissues and cell lines. The binding position between hsa_circ_0053063 and miR-330-3p was confirmed by luciferase reporter assay. Moreover, hsa_circ_0053063 inhibited cell viability, proliferation, and progression of BC through the negative regulation of miR-330-3p. Programmed cell death 4 (PDCD4) is a direct target of miR-330-3p. Besides, the over-expression of miR-330-3p promoted cell progression by directly targeting and regulating PDCD4. Mechanistically, hsa_circ_0053063 activated PDCD4 by targeting miR-330-3p to inhibit BC progression. In conclusion, hsa_circ_0053063 inhibits breast cancer cell proliferation via hsa_circ_0053063/hsa-miR-330-3p/PDCD4 axis, which may provide a new therapeutic target for BC patients.

## INTRODUCTION

Breast cancer (BC) is a major disease that threatens women's health. The incidence (24.2%) and mortality (15.0%) are higher than other cancers worldwide for females based on GLOBOCAN database (2018) [1]. In recent years, more diagnostic and therapeutic methods of BC have been developed. However, the incidence rate and death rate of BC have not decreased [2]. Metastasis, recrudescence, and drug resistance are very common in BC and have caused many relative deaths. Therefore, exploring the molecular mechanism of BC is still a vital task.

Recent studies indicated that non-coding RNAs (ncRNAs) take part in mediating BC genesis and progression [3]. MicroRNAs (miRNAs), such as miR-200b, miR-497, and miR-21, regulate BC-related gene expression mainly through post-transcriptional ways [4–6]. Long non-coding RNAs (lncRNAs) including lncRNA LINK-A and lncRNA POU3F3 affect the genesis, metabolism, proliferation, and apoptosis of BC [7, 8]. Circular RNAs (circRNAs) are novel ncRNAs with closed continuous loops formed by back splicing [9]. The first circRNA was discovered in 1976. At that time, circRNAs were regarded as mis-splicing or by-products of pre-mRNA processing for a long while [10, 11]. Nowadays, a great number of circRNAs have been detected and their biological functions are partly uncovered. The underlying mechanism of circRNAs' functions mainly includes miRNA or protein sponges, enhancer of protein function, protein scaffolding, protein recruitment, and templates for peptides translation [12]. As for tumors, some studies revealed that circRNAs could participate in tumorigenesis and they may be the potential biomarkers and therapeutic targets of cancers [10, 12]. Therefore, the regulatory function of circRNAs in BC is worthy to study.

PDCD4 (Programmed Cell Death 4), was cloned from the human library by Matsuhashi's group in 1997 [13]. Then in 1999, PDCD4 was firstly described as a tumor suppressor by Cmarik's group, which found that PDCD4 could inhibit neoplastic transformation in mouse JB6 cells [14]. The human PDCD4 gene is located on chromosome 10q24 and encoded by 185 amino acids. In recent years, PDCD4 has been found to participate in the oncogenesis and progression of BC cells [15, 16]. Study on enhancing the activity of PDCD4 may bring an advance in the treatment of BC [17].

In this study, we found a highly expressed circRNA in BC, hsa_circ_0053063 and then we exposed it as a tumor suppressor via negative regulating miR-330-3p expression to activate PDCD4. This provided new insight into BC treatment.

## RESULTS

### Characterization of hsa_circ_0053063 in BC cells

**Figure 1:**
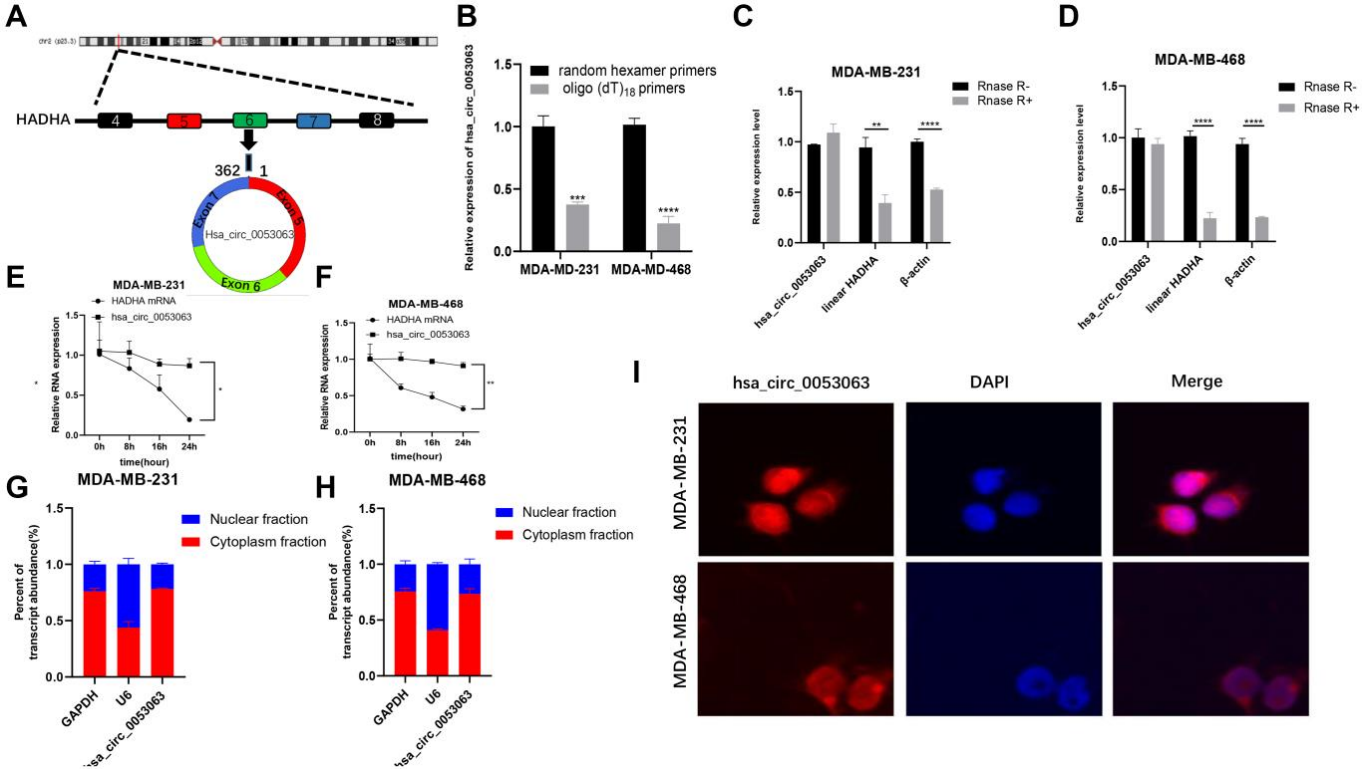
**Characterization of hsa_circ_0053063 in BC cells.** (**A**) Hsa_circ_0053063 is composed of exon 5-7 of gene HADHA. (**B**) qRT-PCR was used to test the expression of hsa_circ_0053063 in MDA-MB-231 and MDA-MB-468 cells with random hexamer or oligo (dT)18 primers. (**C–D**) qRT-PCR analysis revealed the expression of hsa_circ_0053063, linear HADHA, and β-actin in MDA-MB-231 and MDA-MB-468 cells treated with or without RNase R. (**E–F**) Expression of PDCD4 mRNA level and hsa_circ_0053063 in MDA-MB-231 and MDA-MB-468 cells treated with actinomycin D. (**G–H**) Expression levels of GAPDH, U6, and hsa_circ_0053063 in the cell cytoplasmic and nuclear were determined by qRT-PCR in MDA-MB-231 and MDA-MB-468 cells. (**I**) RNA FISH for hsa_circ_0053063, and nuclei was stained with DAPI. (^*^p < 0.05, ^**^p < 0.01, ^***^p < 0.001, ^****^p < 0.0001).

Data from high throughput sequencing chip GSE101123 in GEO (https://www.ncbi.nlm.nih.gov/geo/) indicated that hsa_circ_0053063 is highly expressed in BC tissues than in adjacent normal ones. According to the UCSC (http://genome.ucsc.edu/), hsa_circ_0053063 has a spliced length of 362 bp and is generated from the 5th-7th exon of the gene HADHA, which is located at chr2:26453059-26457223 (Figure 1A). When oligo (dT)18 primers were used, the expression of hsa_circ_0053063 decreased in MDA-MB-231 and MDA-MB-468 cell lines compared with random hexamer primers. This result meant that hsa_circ_0053063 lacked the 3'poly-A tail and had a unique closed-loop structure (Figure 1B). Besides, the total RNA of MDA-MB-231 and MDA-MB-468 cells were treated with Rnase R exonuclease, through qRT-PCR, the results verified that hsa_circ_0053063 has a circular structure in these two cell lines (Figure 1C–1D). To confirm the stability of hsa_circ_0053063, the relative expression of HADHA mRNA level was reduced by about 70% while there was almost no decrease of hsa_circ_0053063 after treatment with actinomycin D for 24 h (Figure 1E–1F). Moreover, qRT-PCR was used to explore the relative expression levels of GAPDH (cytoplasmic control transcripts), U6 (nuclear control transcript), and hsa_circ_0053063 in the cytoplasm and nuclear fractions from MDA-MB-231 and MDA-MB-468 cells. The results indicated that hsa_circ_0053063 was enriched in the cytoplasm (Figure 1G–1H). This is also confirmed by the FISH analysis (Figure 1I).

### Hsa_circ_0053063 is highly expressed in BC and acts as an antioncogene in BC cells

**Figure 2:**
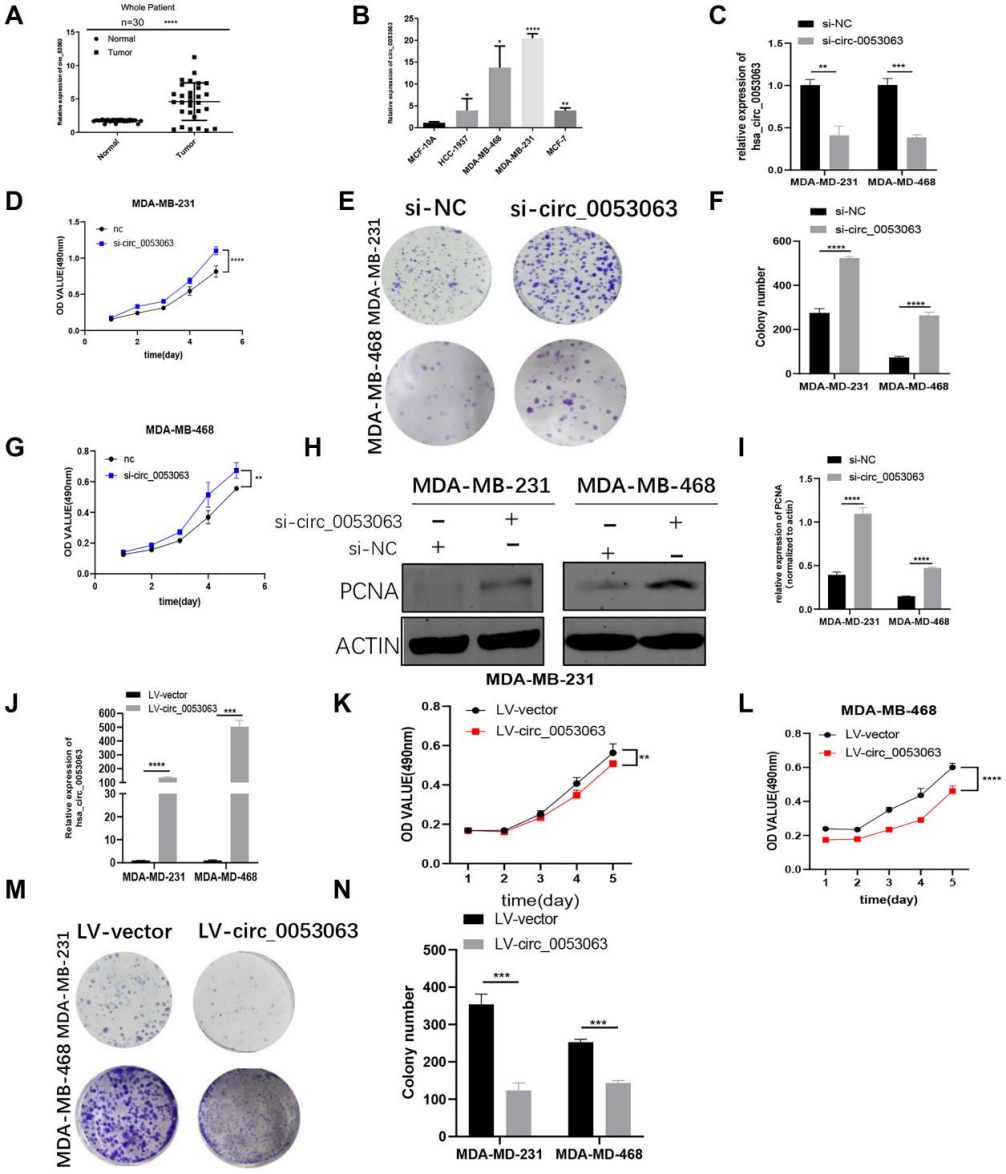
**Hsa_circ_0053063 is highly expressed in BC and acts as an antioncogene in BC cells.** (**A**) qRT-PCR analysis revealed the expression of hsa_circ_0053063 in BC tissues and adjacent normal tissues. (**B**) Relative expression of hsa_circ_0053063 in BC cell lines compared with normal breast epithelial cell line MCF-10A. (**C**) qRT-PCR analysis revealed the transfection efficiency of hsa_circ-0053063 specific siRNA. (**D–G**) Effect of siRNA targeting hsa_circ_0053063 on MTT assays and colony formation in MDA-MB-231 and MDA-MB-468 cells. (**H–I**) Western blot analysis demonstrated the effect of hsa_circ_0053063 siRNAs on PCNA in MDA-MB-231 and MDA-MB-468 cell lines. (**J**) qRT-PCR revealed the overexpression efficiency of LV-circ_0053063 in MDA-MB-231 and MDA-MB-468 cell lines. (**K–N**) Effect of LV-circ_0053063 on proliferation in MDA-MB-231 and MDA-MB-468 cell lines by MTT assay and colony formation assays. (^*^p < 0.05, ^**^p < 0.01, ^***^p < 0.001, ^****^p < 0.0001).

qRT-PCR was used to measure the relative expression of hsa_circ_0053063 in 30 pairs of BC tissues and normal tissues, which results demonstrated that hsa_circ_0053063 was increased in BC tissues (24/30, 80%) (Figure 2A). Moreover, compared with MCF-10A, the relative expression of hsa_circ_0053063 was also increased in 4 BC cell lines (Figure 2B). Given the results above, we chose MDA-MB-231 and MDA-MB-468 cell lines for subsequent experiments. To explore the function of hsa_circ_0053063 in BC, we used siRNA (si-circ_0053063) to inhibit hsa_circ_0053063 function in the MDA-MB-231 and MDA-MB-468 cells. Also, we used qRT-PCR to confirm the transfection efficiency of this specific siRNA (Figure 2C). Both MTT and colony formation assays showed that si-circ_0053063 increased cell proliferation in the MDA-MB-231 and MDA-MB-468 cells (Figure 2D–2G). Western blot analysis demonstrated that PCNA, a proliferation marker, could be raised by si-circ_0053063 (Figure 2H–2I). Besides, we stably overexpressed hsa_circ_0053063 in these two cell lines (Figure 2J). MTT and colony formation assays were used to prove over-expression hsa_circ_0053063 could inhibit BC cell proliferation (Figure 2K–2N). To investigate whether there is a connection between the expression of hsa_circ_0053063 and multiple clinic pathological variables, these 30 patients' clinical data were used for analysis. Results showed that hsa_circ_0053063 high expression was only positively related to the age of these patients. Due to its small sample size, this connection needs to be further verified (Table 1).

**Table 1:** The relationship between the expression of has_circ_0053063 and various clinicopathological variables.

**Patients Characteristics**	**Total**	**hsa_circ_0053063 expression**		
		**High (*N = 25)***	**Low (*N = 5)***	*P* value*
Age				0.0455*
<60	18	17	1	
≥60	12	8	4	
TNM stage				0.33669
I and II	26	21	5	
III and IV	4	4	0	
Lymph node metastasis				0.23556
negative	19	17	2	
positive	11	8	3	
Tumor size(cm)				0.51248
≤2	14	11	3	
>2	16	14	2	
Recrudescence				0.71229
No	22	18	4	
Yes	8	7	1	

### Hsa_circ_0053063 inhibits BC tumor growth *in vivo*

**Figure 3:**
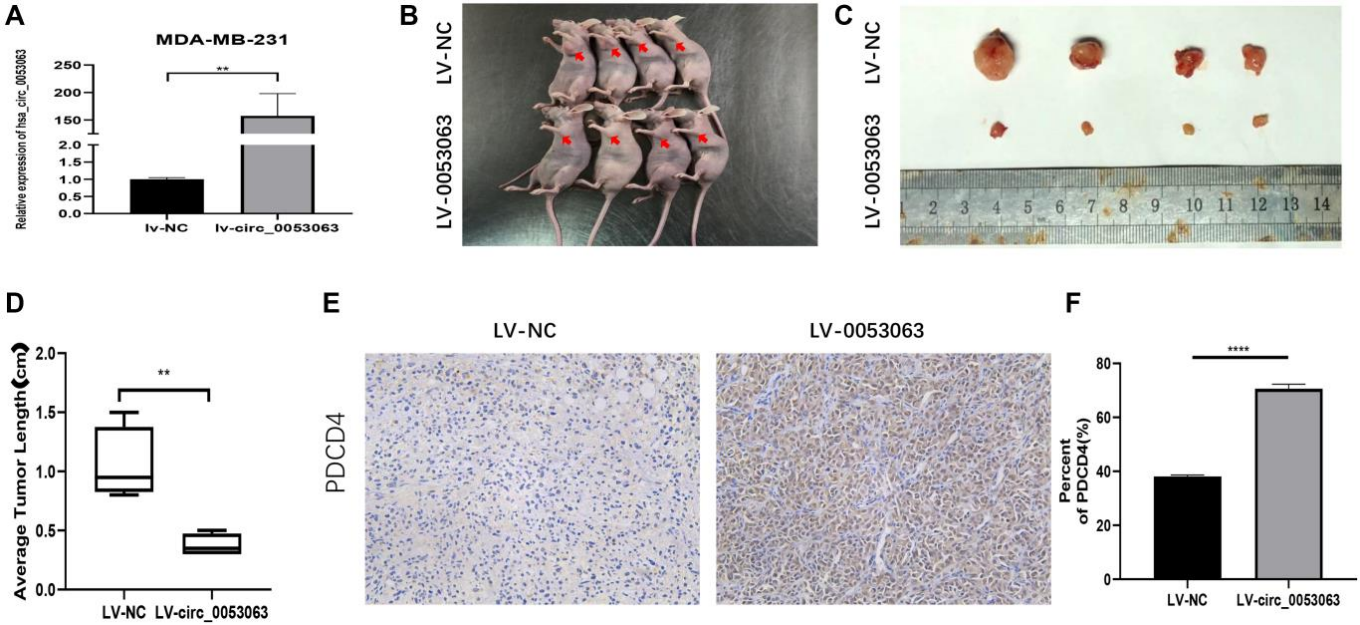
**Hsa_circ_0053063 inhibits BC tumor growth *in vivo*.** (**A**) Hsa_circ_0053063 overexpression efficiency in MDA-MB-231 cells was affirmed by qRT-PCR. (**B**) The nude mice injected into the fat pad of the right second breast. (**C–D**) The mice tumors were photographed and measured. (**E–F**) IHC assay revealed PDCD4 protein expression in the over-expression group and NC group. (^*^p < 0.05, ^**^p < 0.01, ^***^p < 0.001, ^****^p < 0.0001).

The xenograft tumor assay was performed to verify the function of hsa_circ_0053063 *in vivo*. MDA-MB-231 cells were stably transfected with lv-circ_0053063 or lv-NC. The expression of hsa_circ_0053063 was verified by qRT-PCR (Figure 3A). The mice were injected with these two group cells (Figure 3B). The mice tumors were photographed, measured, and weighed. The mice tumors of the lv-circ_0053063 group are significantly smaller than the control group (Figure 3C–3D). Immunohistochemistry assay revealed PDCD4 protein expression was higher in the hsa_circ_0053063 overexpression group (Figure 3E–3F). Through the results above, we proved that hsa_circ_0053063 can inhibit BC tumor growth.

### Hsa_circ_0053063 inhibited the expression of miR-330-3p

**Figure 4:**
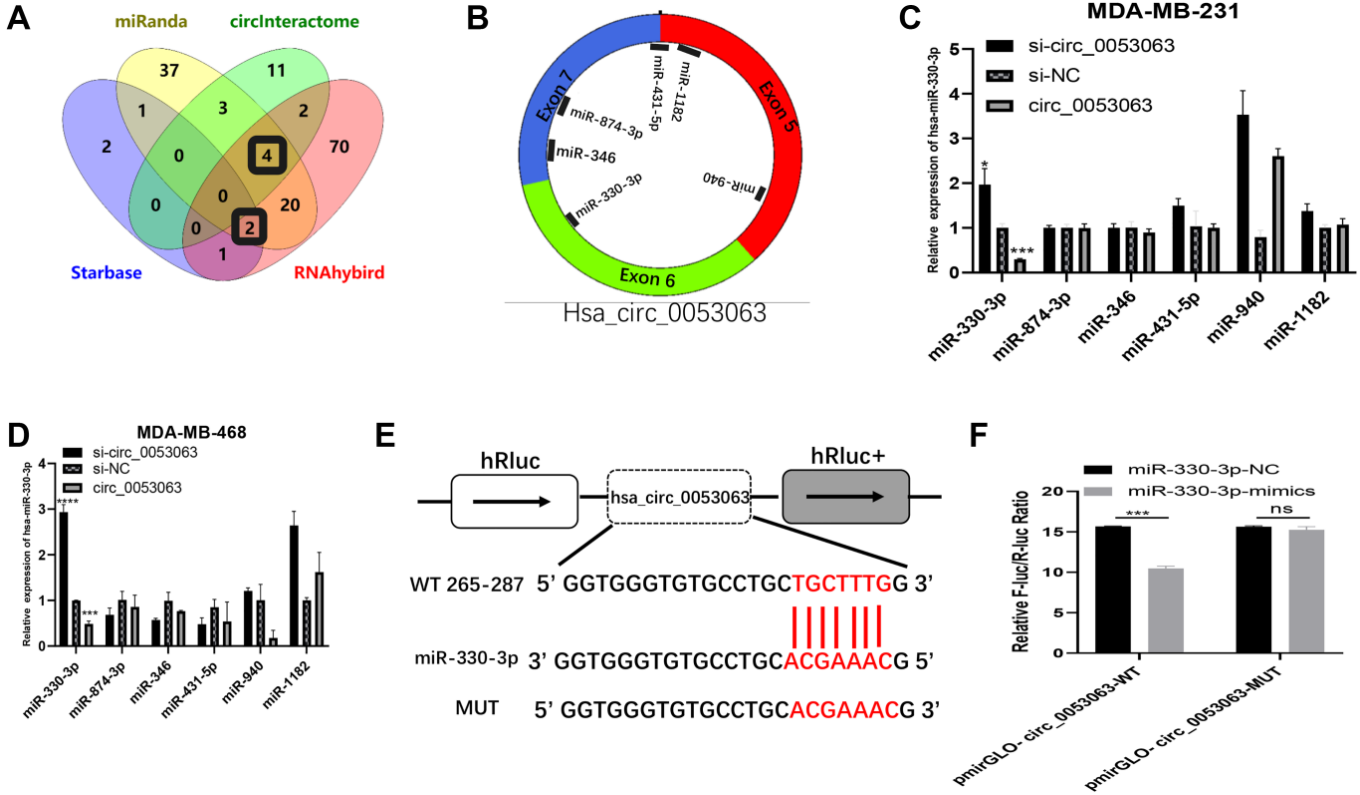
**Hsa_circ_0053063 inhibited the expression of miR-330-3p.** (**A–B**) Databases were employed to predict potential miRNA target of hsa_circ_0053063 and Venn diagram showed the target miRNAs from intersections (miR-330-3p, miR-346, miR-431-5p, miR-874-3p, miR-1182). (**C–D**) qRT-PCR was used to verify which miRNAs can be regulated by hsa_circ_053063 in MDA-MB-231 and MDA-MB-468 cell lines. (**E**) Predicted binding sites of hsa_circ_0053063 for miR-330-3p. (**F**) Dual-luciferase reporter assay evaluating the interaction between wild-type hsa_circ_0053063 + miR-330-3p reporter compared with mutant hsa_circ_0053063+ miR-330-3p reporter. (^*^p < 0.05, ^**^p < 0.01, ^***^p < 0.001, ^****^p < 0.0001).

During the past several years, there have been many studies on how circRNAs work as miRNA sponges to realize the function. In consideration of hsa_circ_0053063 is mainly located in the cytoplasm, we assumed that hsa_circ_0053063 inhibits breast cancer cell proliferation through realizing function by acting as a ceRNA. Four databases, Starbase (https://web.archive.org/web/20110222111721/http://starbase.sysu.edu.cn/), CircInteractome (https://circinteractome.nia.nih.gov/), Miranda and RNAhybird (http://bibiserv.techfak.uni-bielefeld.de/rnahybrid/) were employed to predict potential miRNA binding target of hsa_circ_0053063. We integrated these data into a Wynn diagram and chose mRNAs from intersections of at least three databases, in the end, 6 miRNAs (miR-874-3p, miR-431-5p, miR-18182, miR-940, miR-346, miR-330-3p) were selected (Figure 4A–4B). Then, qRT-PCR was used to verify whether miRNAs' expression can be inhibited by hsa_circ_0053063. Results indicated that both in MDA-MB-231 and MDA-MB-468 cell lines, only miR-330-3p presented a negative correlation with hsa_circ_0053063, which suggested that hsa_circ_0053063 acts as a ceRNAs to bind miR-330-3p (Figure 4C–4D). Negatively correlations between the expression of hsa_circ_0053063 and miR-330-3p were found in 30 BC samples (Supplementary Figure 1A). As predicted by CircInteractome, hsa_circ_0053063 contained the putative bind sites for miR-330-3p (Figure 4E). We confirm that hsa_circ_0053063 could directly bind to miR-330-3p through dual-luciferase assays. Overexpression of miR-330-3p decreased the luciferase activity of wild-type hsa_circ_0053063, while it did not have any influence on the luciferase activity of mutation-type hsa_circ_0053063 (Figure 4F). Therefore, these data indicated that hsa_circ_0053063 directly binds to miR-330-3p.

### MiR-330-3p highly expresses and acts as an oncogene in BC cells

**Figure 5:**
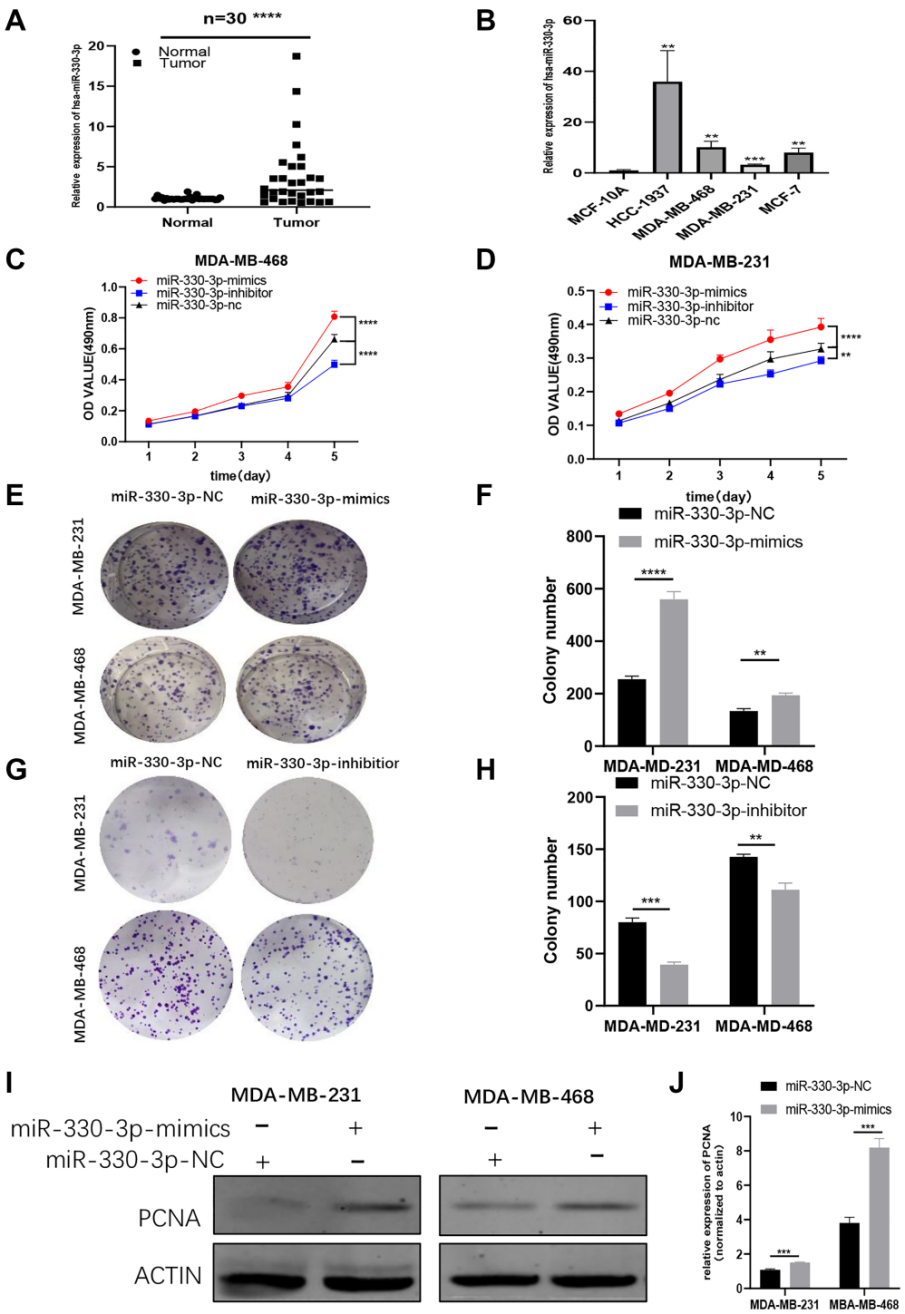
**MiR-330-3p highly expresses and acts as an oncogene in BC cells.** (**A–B**) QRT-PCR assays revealed miR-330-3p expression in BC patient tissues and BC cell lines. (**C–H**) MTT assays and colony formation assays were performed in MDA-MB-231 and MDA-MB-468 cell lines to determine the effect of miR-330-3p-inhibitor and miR-330-3p-mimics on proliferation. (**I–J**) Effect of miR-330-3p-mimics on proliferation in MDA-MB-231 and MDa-MB-468 cells by western blot. (^*^p < 0.05, ^**^p < 0.01, ^***^p < 0.001, ^****^p < 0.0001).

To further explore the function of miR-330-3p in BC, the miR-330-3p expression level was tested by using qRT-PCR in BC cell lines. A significant trend of high expression was observed in BC patient tissues and cell lines, which implied that miR-330-3p may act as an oncogene in BC cells (Figure 5A–5B). To verify the function of miR-330-3p, MTT assays and colony formation assays were performed in MDA-MB-231 and MDA-MB-468 cell lines. The results indicated that miR-330-3p mimics could promote the proliferation of BC cells while miR-330-3p inhibitor was on the opposite (Figure 5C–5H). Also, western blot results showed that PCNA expression was raised by miR-330-3p mimics (Figure 5I–5J). These results demonstrated that that miR-330-3p could accelerate the proliferation function of BC cells.

### PDCD4 served as a molecular target of miR-330-3p

**Figure 6:**
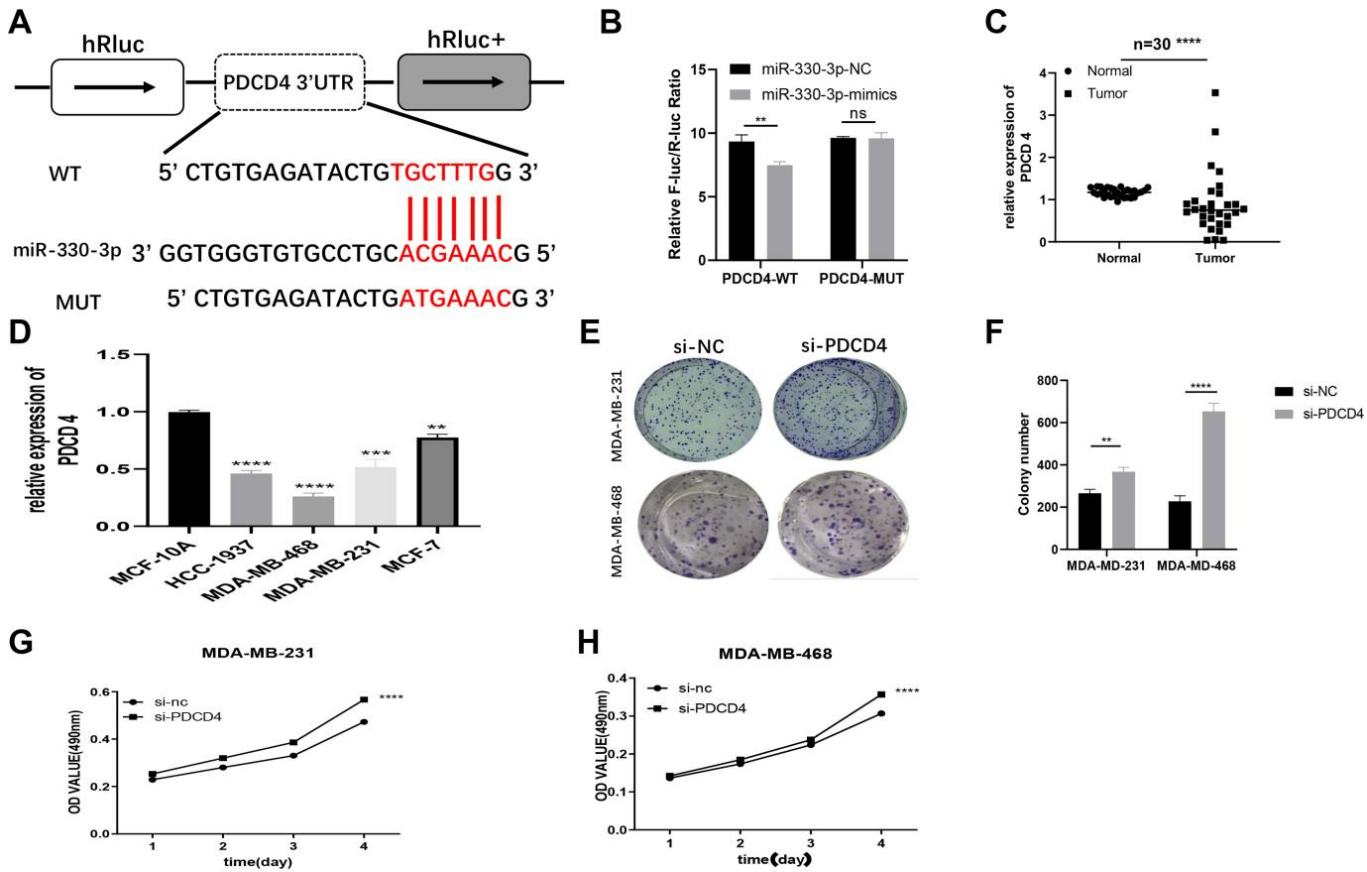
**PDCD4 served as a molecular target of miR-330-3p.** (**A**) Putative complementary sites within PDCD4 and R-330-3p predicted by TargetScan. (**B**) Dual-luciferase reporter assay evaluates the interaction between wild-type PDCD4 + miR-330-3p reporters compared with mutant PDCD4+ miR-330-3p reporter. (**C–D**) The expression levels of PDCD4 were tested in both BC tissues and cell lines through QRT-PCR. (**E–H**) Effect of siRNA targeting PDCD4 on colony formation and MTT assays in MDA-MB-231 and MDA-MB-468 cell lines. (^*^p < 0.05, ^**^p < 0.01, ^***^p < 0.001, ^****^p < 0.0001).

We found PDCD4 was a putative target of miR-330-3p by TargetScan (http://www.targetscan.org/vert_71/). It has been proved that PDCD4 is a target gene of miR-330-3p in human esophageal cancer [18]. Results from qRT-PCR demonstrated that PDCD4 presented with low expression in BC patient tissues and BC cell lines (Supplementary Figure 1B–1C). So we conjectured that hsa_circ_0053063 inhibits BC cell proliferation through targeting the miR-330-3p/PDCD4 axis in BC. Through dual-luciferase assays, we confirmed that PDCD4 was the direct target of miR-330-3p (Figure 6A). The wild and mutant dual-luciferase reporter plasmids of PDCD4 were constructed, and the data indicated that miR-330-3p mimics reduced the luciferase activity of the PDCD4-WT luciferase reporter but not that of mutant ones (Figure 6B). Then, PDCD4 expression was tested in both BC tissues and cell lines through qRT-PCR, which results demonstrated that PDCD4 was low-expressed in BC (Figure 6C–6D). Furthermore, to verify the function of PDCD4 in BC, PDCD4 siRNA was used to transfect BC cell lines. Through MTT and colony formation assays, the results indicated that PDCD4 could inhibit cell proliferation in BC (Figure 6E–6H).

### Hsa_circ_0053063 activated PDCD4 through targeting miR-330-3p

**Figure 7:**
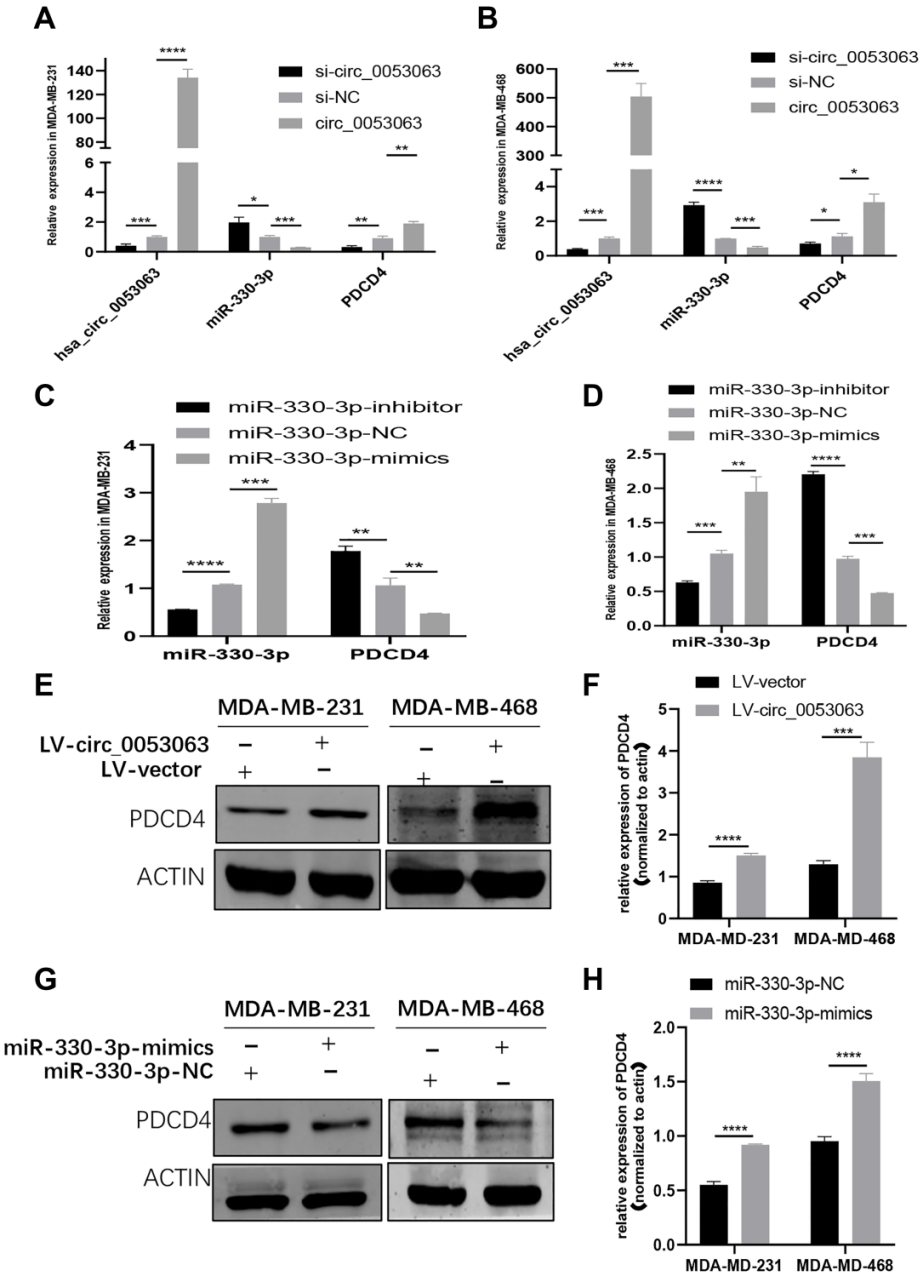
**Hsa_circ_0053063 activated PDCD4 through targeting miR-330-3p.** (**A–B**) qRT-PCR analysis revealed the expression of PDCD4 after transfecting miR-330-3p mimics, hsa_circ_0053063 siRNA, and LV-circ-0053063 in MDA-MB-231 and MDA-MB-468 cells. (**C–D**) qRT-PCR analysis revealed the expression of PDCD4 mRNA in MDA-MB-231 and MDA-MB-468 cells treated with miR-330-3p mimics and inhibitors. (**E–F**) Western blot assays were employed to detect the protein levels of PDCD4 in MDA-MB-231 and MDA-MB-468 cell lines transfected with lv-circ_0053063. (**G–H**) Western blot assays were employed to detect the protein levels of PDCD4 in MDA-MB-231 and MDA-MB-468 cell lines transfected with miR-330-3p mimics. (^*^p < 0.05, ^**^p < 0.01, ^***^p < 0.001, ^****^p < 0.0001).

To further explore whether hsa_circ_0053063 could activate the expression of PDCD4 or not. First, the relative expression of miR-330-3p and PDCD4 were detected by qRT-PCR in MDA-MB-231 and MDA-MB-468 cells infected by si-circ_00053063 and LV-circ_0053063 (Figure 7A–7B). The results showed that si-circ_0053063 could increase the expression of miR-330-3p meanwhile decrease the expression of PDCD4. In the meanwhile, lv-circ_0053063 could do the opposite. On the other hand, qRT-PCR results revealed that miR-330-3p could reduce the expression level of PDCD4 mRNA, while the miR-330-3p inhibitor got opposite results (Figure 7C–7D). To confirm that hsa_circ_0053063 and miR-330-3p could influence PDCD4 on the protein level, western blot assays were employed. The results demonstrated that when transfected with lv-circ_0053063, the protein levels of PDCD4 were increased, while the results were, on the contrary when transfected with miR-330-3p mimics (Figure 7E–7H). Collectively, hsa_circ_0053063 could activate PDCD4 through targeting miR-330-3p.

### Hsa_circ_0053063 generated the anti-oncogenic function in BC cells via the regulation of miR-330-3p/PDCD4 axis

**Figure 8:**
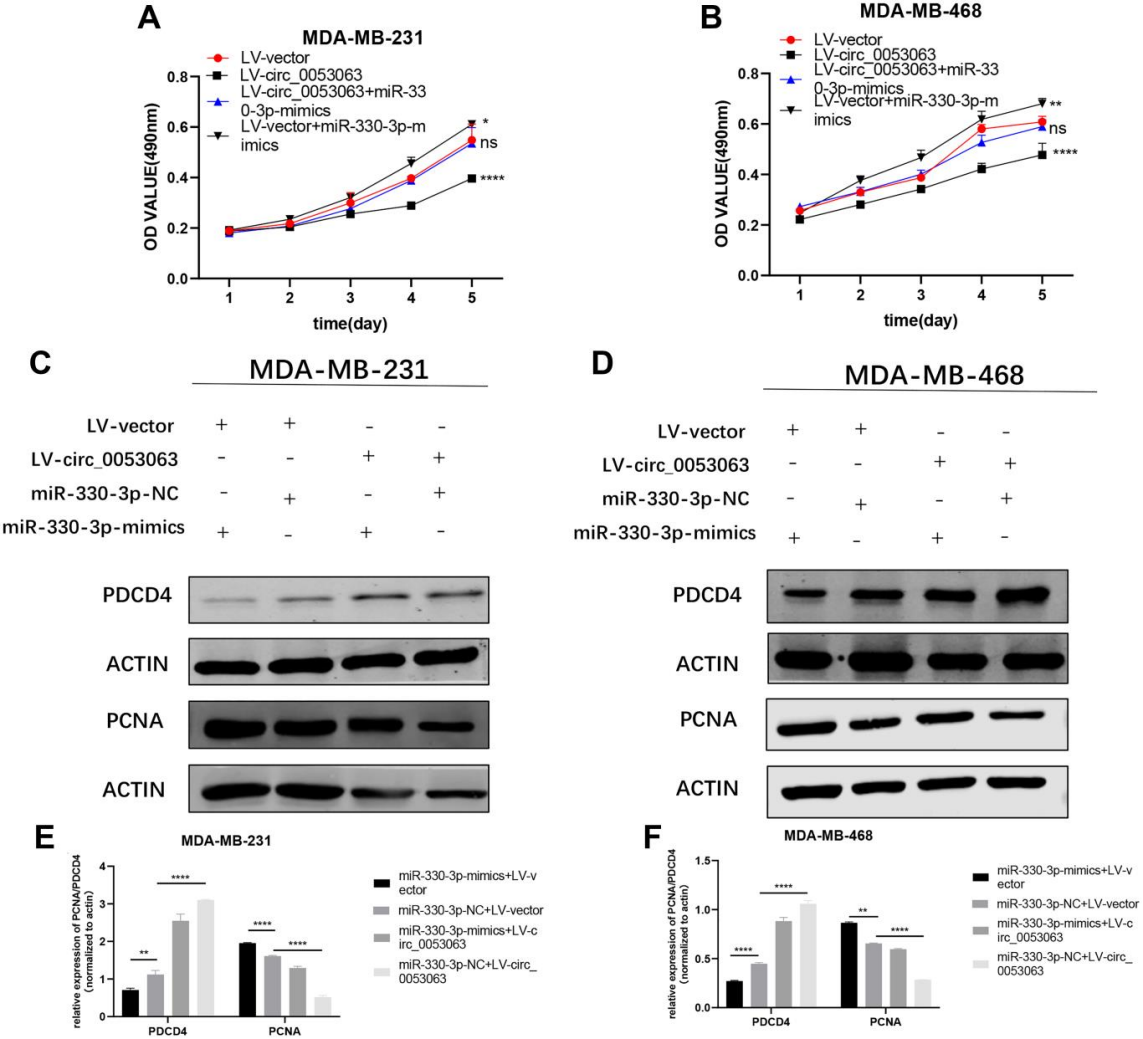
**Hsa_circ_0053063 generated the anti-oncogenic function in BC cells via the regulation of miR-330-3p/PDCD4 axis.** (**A–B**) Effect of LV-circ_0053063 and miR-330-3p mimics on proliferation in MDA-MB-231 and MDA-MB-468 cell lines by MTT assay. (**C–F**) Western blot assay revealed the protein level of PDCD4 transfected with hsa_circ_0053063 and miR-330-3p mimics in MDA-MB-231 and MDA-MB-468 cell lines (^*^p < 0.05, ^**^p < 0.01, ^***^p < 0.001, ^****^p < 0.0001).

To explore the biological function of the hsa_circ_0053063-miR-330-3p-PDCD4 axis and verify that hsa_circ_0053063 could increase PDCD4 protein level by absorbing miR-330-3p in BC, we performed rescue assays in MDA-MB-231 and MDA-MB-468 cell lines. According to the results of MTT assays, the hsa_circ_0053063-induced negative effects on proliferation were relieved after transfected with miR-330-3p mimics (Figure 8A–8B). Moreover, according to western blot assays, the increased protein level of PDCD4 induced by hsa_circ_0053063 was alleviated by miR-330-3p (Figure 8C–8F). The PCNA protein level also indicated that miR-330-3p mimics could reveal the decreased cell proliferation of BC cells induced by hsa_circ_0053063. In a word, we confirmed that hsa_circ_0053063 generated the anti-oncogenic function in BC cells via the miR-330-3p/PDCD4 axis regulation.

### Hsa_circ_0053063 inactivated P53 through miR-330-3p/PDCD4 axis

**Figure 9:**
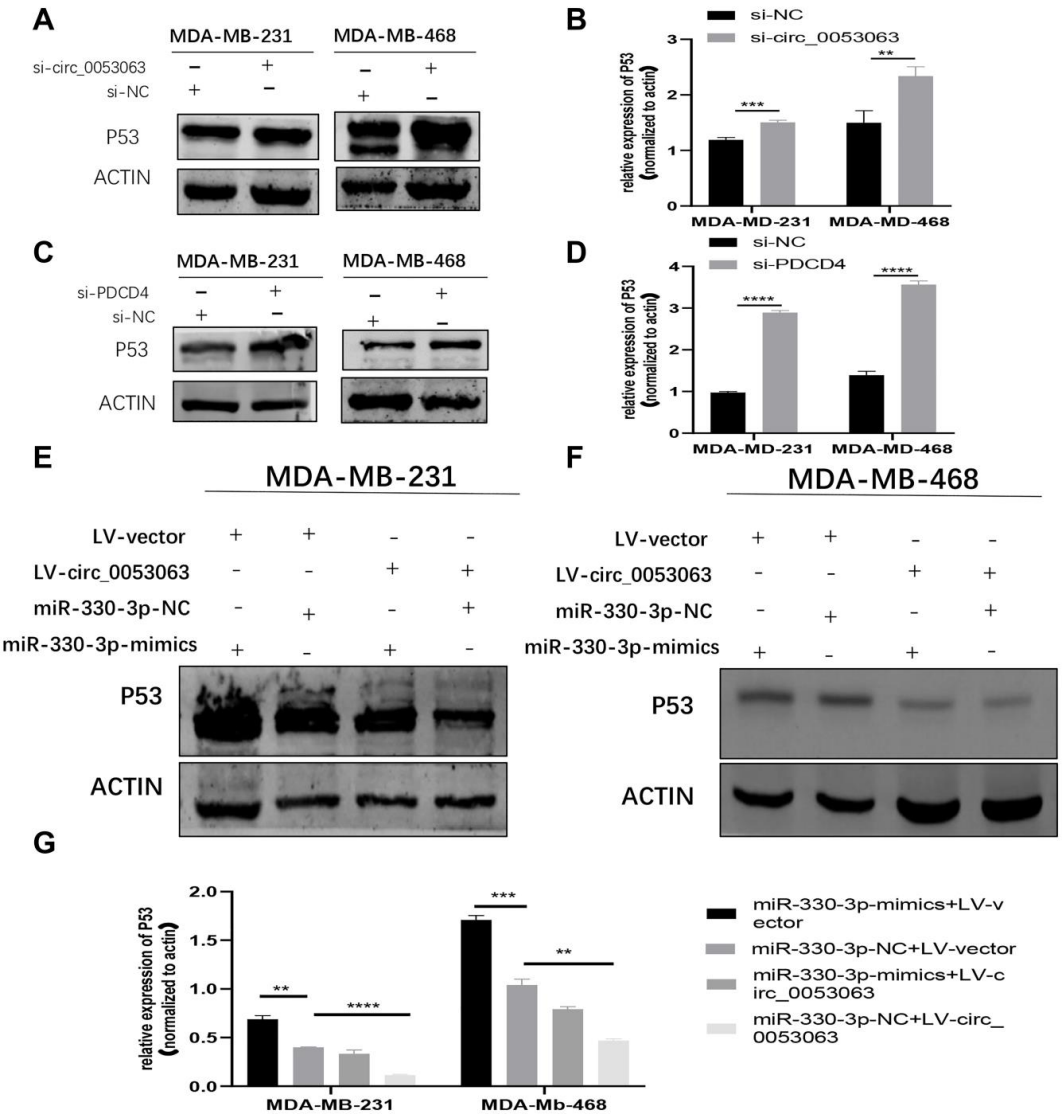
**Hsa_circ_0053063 inactivated P53 through miR-330-3p/PDCD4 axis.** (**A–B**) Western blot assay revealed the P53 protein level in MDA-MB-231 and MDA-MB-468 cells treated with hsa_circ_0053063 siRNA. (**C–D**) Western blot assay revealed the P53 protein level in MDA-MB-231 and MDA-MB-468 cell lines transfected with PDCD4 siRNA. (**E–G**) Western blot assay revealed the protein level of P53 in MDA-MB-231 and MDA-MB-468 cell lines transfected with hsa_circ_0053063 and miR-330-3p mimics. (^*^p < 0.05, ^**^p < 0.01, ^***^p < 0.001, ^****^p < 0.0001).

PDCD4 can suppress the translation of P53 mRNA [19]. Therefore, western blot was employed to explore whether hsa_circ_0053063 could inactivate P53 through miR-330-3p/PDCD4 axis. The results showed that si-circ_0053063 could induce P53 protein enrichment (Figure 9A–9B). PDCD4 siRNA could also accrete the P53 protein level (Figure 9C–9D). Further investigation showed that the decreased protein level of P53 induced by hsa_circ_0053063 can be alleviated by miR-330-3p mimics which indicated that hsa_circ_0053063 inactivated P53 through miR-330-3p/PDCD4 axis (Figure 9E–9G).

### The mechanism diagram shows how hsa_circ_0053063 inhibits BC cells proliferation by miR-330-3p as a ceRNA

**Figure 10:**
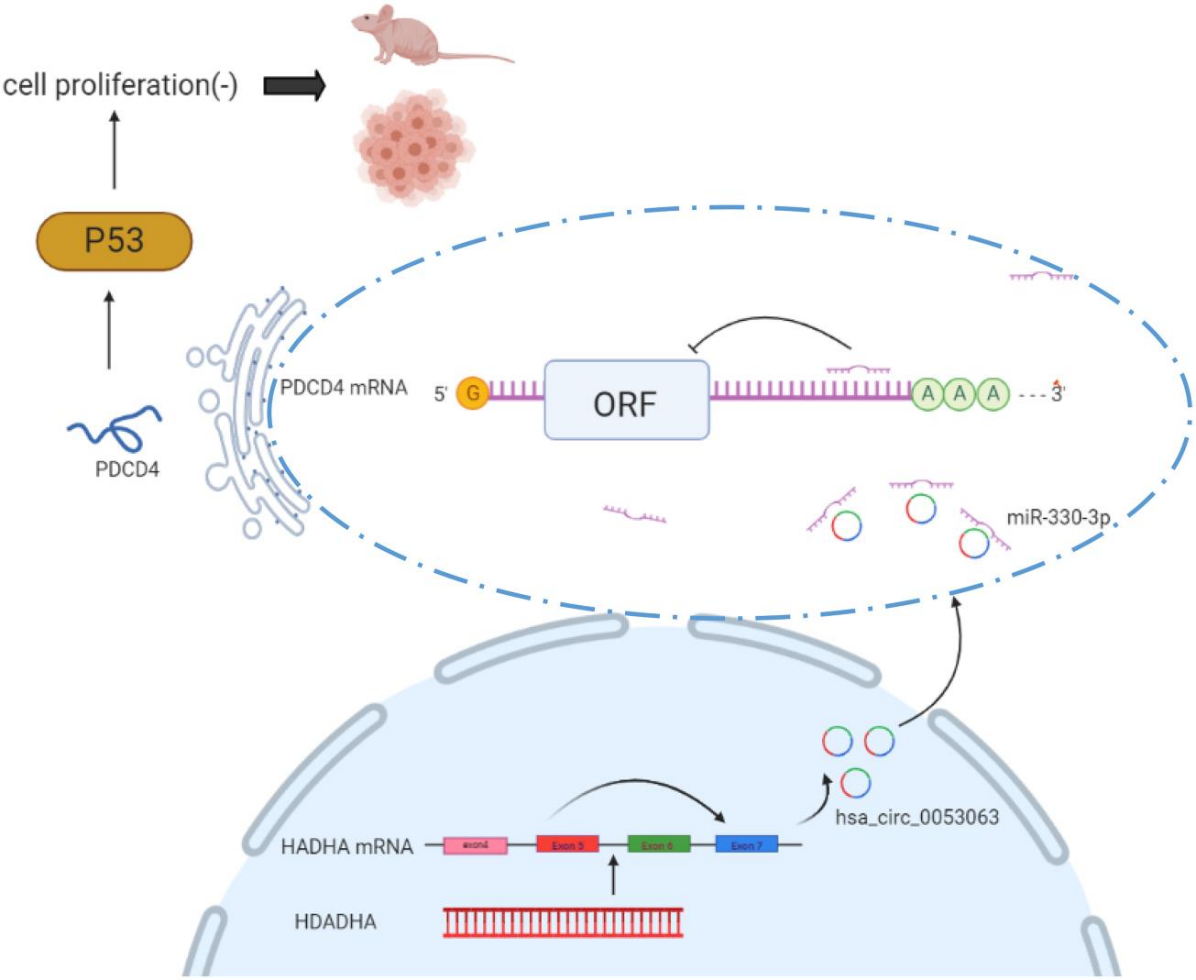
**Mechanism diagram shows how hsa_circ_0053063 inhibits BC cell proliferation by miR-330-3p as a ceRNA.**

Taken together, hsa_circ_0053063 inhibits the expression of miR-330-3p, thus it could activate one of miR-330-3p's downstream genes PDCD4 through targeting miR-330-3p. So hsa_circ_0053063 generated the anti-oncogenic function in BC cells via regulating miR-330-3p/PDCD4 axis. The mechanism diagram illustrates how this axis works and this study provided a new clinical diagnostic and therapeutic target of BC (Figure 10).

## DISCUSSION

First found in viruses, then proved to be presented in eukaryotic cells, circular RNAs (circRNAs) are a species of non-coding RNAs with covalently closed-loop structure, which provides its stability characteristics [11, 20]. Studies from the past several decades have revealed the function of part of circRNAs so that plentiful circRNAs have been uncovered to be vital in biological progress and may participate in the development of cancers [21, 22]. CircRNAs have no 5' caps and 3' ploy-A tails causing their stability and long half-life [23]. Based on the above advantages, circRNAs may serve as better diagnostic biomarkers than other ncRNAs.

More and more evidence has been put forward on how circRNAs work as a miRNA sponge to execute its function to influence tumor progression [24]. For example, the miR-29b family was decoyed by circRNA_100290 to regulate CDK6 expression [25]. Also, circRNAs as a regulatory factor and a valuable diagnostic marker of BC have been proved. For instance, circ_0001667 promoted BC cell proliferation and survival via regulating TAZ to influence the hippo pathway [26], hsa_circ_0001982 promoted BC cell carcinogenesis through diminishing miR-143 [27].

In this study, data from high throughput sequencing chip GSE101123 in the GEO database indicated that a circRNA, hsa_circ_0053063 may have a higher expression in BC tissues. Then qRT-PCR was performed to confirm that hsa_circ_0053063 was highly expressed in BC tissues as well as in BC cell lines. Moreover, the expression has a connection with the age of BC patients, suggesting that hsa_circ_0053063 may conduct as a diagnostic biomarker for aged BC patients. Through functional experiments, we found that over-expressed hsa_circ_0053063 significantly decreased cell viability and proliferation, by the contrary, si-circ_0053063 increased cell viability and proliferation. Moreover, we confirmed this *in vivo* through mice experiments. All results above demonstrated that hsa_circ_0053063 served as a cancer-suppressor in BC progression. MiR-330-3p has been predicted and validated as a target of hsa_circ_0053063. Studies have revealed the tumor accelerator function of miR-330-3p in BC [28]. To explore the underlying mechanism between hsa_circ_0053063 and miR-330-3p, we performed functional experiments and the results proved the correlations between them. Firstly, hsa_circ_0053063 was negatively correlated with miR-330-3p. Secondly, miR-330-3p mimics significantly increased cell viability and proliferation, indicating the ability of miR-330-3p to promote BC cell progression. Lastly, rescue experiments implied that hsa_circ_0053063-induced decreasing cell proliferation could be recovered by miR-330-3p mimics, demonstrating that hsa_circ_0053063 realized its anti-oncogene function by targeting miR-330-3p. However, the deep regulation mechanism under this hsa_circ_0053063/miR-330-3p axis is still unknown and needs to be further studied. Based on the researches before, PDCD4 is a tumor suppressor [29, 30]. In BC, PDCD4 has been proved to inhibit the BC cell invasion [31, 32]. Importantly, PDCD4 is predicted to be a putative gene of miR-330-3p by using databases. Therefore, hsa_circ_0052063 influences the regulation of PDCD4 through sponging miR-330-3p, thus regulating cell biological functions of BC. qRT-PCR was employed to test the expression of PDCD4 in MDA-MB-231 and MDA-MB-468 cells transfected with si-circ_0053063 and LV-circ_0053063. Results indicated that LV-circ_0053063 could significantly enhance the expression level of PDCD4, while si-circ_0053063 was, on the contrary, demonstrated that hsa_circ_0052063 played a vital role in PDCD4 regulation. The function experiment showed the inhibitory effect of PDCD4 on the proliferation of BC. From the western blot assay, we proved that miR-330-3p promoted cell proliferation by inhibiting PDCD4. In general, the present study demonstrated that hsa_circ_0053063 was treated as a miR-330-3p sponge, and executed its function by removing the inhibitory effect of miR-330-3p on its target PDCD4, finally, regulating the expression of PDCD4.

P53 has been considered as an identified translational target of PDCD4 [19]. Further studies have proved that hsa_circ_0053063 could inhibit the P53 protein level through the miR-330-3p/PDCD4 axis.

Therefore, the discovery of hsa_circ_0053063 provides a PDCD4 accelerator, which may be a new diagnostic target and beneficial for BC patients in the further.

## CONCLUSIONS

In conclusion, we found a novel circRNA that is highly expressed in BC, hsa_circ_0053063. We revealed its underlying mechanism as a tumor suppressor to activate PDCD4 via serving as a sponge of miR-330-3p. These experiential results imply a new potential biomarker or therapeutic target for BC.

## MATERIALS AND METHODS

### Human BC tissue samples

30 pairs of tumor tissues and matched normal tissues from BC patients undergoing surgery were obtained from the Department of Breast and Thyroid Surgery of Shanghai Tenth People's Hospital (Shanghai, China). The clinical samples were immediately stored in liquid nitrogen after surgery. All patients had received no anti-tumor therapy before and the details of their clinical information were available. Both tumor tissue and adjacent non-tumor tissue were confirmed by specialized pathologists. Consents were given and this study was approved by the Institution Ethics Committees of Shanghai Tenth People's Hospital.

### Cell culture and actinomycin D treatment

MCF-10A cells, the human normal breast cells, were purchased from Zhongqiaoxinzhou Biotech (Shanghai, China) and cultured in Mammary Epithelial Cell Medium (MEpiCM, ScienCell, Research Laboratories, Carlsbad, CA, USA). BC cell lines including MDA-MB-231, MCF-7, HCC-1937, SKBR3, MDA-MB-468 were purchased from the Chinese Academy of Sciences (Shanghai) and cultured in Dulbecco's Modified Eagle’s Medium (DMEM) (Gibco, USA) adding 10% Fetal Bovine Serum (FBS) (Gibco), penicillin (100 U/ml) and streptomycin (100 μg/ml) (Enpromise, China). The cell cultural condition of the cell incubator was set at 37°C with 5% CO2. 2 μg/ml actinomycin D (Millipore, Billerica, MA, USA) was added to the DMEM to inhibit transcription of MDA-MB-231 and MDA-MB-468 cells for 0 h, 8 h, 16 h, and 24 h.

### Cell transfection and lentivirus transduction

Si-RNA specifically targeting hsa_circ_0053063 (si-circ_0053063), and si-RNA negative control (si-NC) were purchased from IBSBio (Shanghai, China). MiR-330-3p-mimics, inhibitor, and miRNA negative control (miR-NC) were purchased from RiboBio (Guangzhou, China). PDCD4 siRNA was synthesized by Integrated Biotech Solutions (Shanghai, China). Liposomal transfection reagent (Yeasen, Shanghai, China) was used for cell transfection. MDA-MB-231 and MDA-MB-468 cells were transfected with reagents mentioned above, according to the manufacturer's instructions. Plasmid extraction was finished by DNA Midiprep Kits (Qiagen, Hilden, Germany). A lentivirus plasmid containing circ_0053063 was assembled by ZORIN (Shanghai, China).

### RNA extraction, reverse transcription and qRT-PCR

Nuclear and cytoplasmic RNA was extracted individually by the PARIS™ Kit (Invitrogen, Carlsbad, CA, USA). Total RNA of cells and tissues was extracted with TRIzol reagent (Invitrogen, Carlsbad, CA, USA). The RNA concentration and purity were assessed with a Nanodrop 2000 spectrophotometer (Thermo Fisher Scientific, Inc.). Reverse transcription was applied to obtain cDNA using HiScript III RT SuperMix kit (Vazyme Biotech, Nanjing, China). qRT-PCR was conducted on a 7900HT Fast RT-PCR instrument (Applied Biosystems, Singapore). Hieff^®^ qPCR SYBR^®^ Green Master Mix, the reagent for qRT-PCR, was purchased from Yeasen (Shanghai, China). Primers were produced by Sangon Biotech (Shanghai, China). 18S rRNA, U6 snRNA, and β-Actin (ACTB) were regarded as internal calibrators for hsa_circ_0053063, miRNA, and mRNA respectively. Primers used in this article are presented in Supplementary Table 1. The results of qRT-PCR were analyzed for relative quantitation using the 2-ΔΔCT method.

### RNase R resistance analysis of circRNAs

Total RNA from MDA-MB-231 and MDA-MB-468 cell lines were treated with RNase R (4 U/mg, Epicenter) according to the manufacturer’s instruction and detected by the qRT-PCR assay.

### MTT assay

About 2000 transfected MDA-MB-231 and MDA-MB-468 cells were planted into 96-well-plates per well. The MTT assay kit (Sangon, Shanghai, China) was used to determine cell viability at 24 h, 48 h, 72 h, and 96 h. The crystal was dissolved in 150 μl dimethylsulfoxide (DMSO, Sangon, Shanghai, China) after 4-hours-incubation together with MTT reagent and the optical density (OD) value was measured at 490 nm using a microplate spectrophotometer (BioTek, Vermont USA).

### Colony formation assay

Transfected MDA-MB-231 and MDA-MB-468 cells were incubated for 10–14 days after seeding into 6-well-plates (500 cells per well). When the cell colonies were visible, the plates were washed twice with phosphate-buffered saline (PBS, Sangon, Shanghai, China). The washed cell colonies were then fixed in 95% ethanol and stained with 0.1% crystal violet. Colony number was counted immediately after taking photographs.

### Dual-luciferase reporter assay

PmirGLO-hsa_circ_0053063 and pmirgGLO-PDCD4 mutant and wild-type reporter plasmids were purchased from Integrated Biotech Solutions (Shanghai, China). 293T cells were planted in 24-well plates. When the confluency of the cells reached 80%, these plasmids were transfected as the transfection procedures used as above. After 24 hours, Dual-Luciferase Reporter Assay (Promega, Madison, WI, USA) was used to detect firefly and Renilla luciferase activities. Finally, the ratio of firefly/Renilla was calculated.

### Protein extraction and western blotting assay

Total proteins of cells were extracted by RIPA buffer (Beyotime, Shanghai, China) on the ice. The supernatants were obtained after centrifuging at 4ºC, 12000 rpm for 30 min. The bicinchoninic acid (BCA) protein assay kit (Beyotime, Shanghai, China) was used to determine the concentration of proteins. The 6 × sodium dodecyl sulfate (SDS) loading buffer was added into proteins and the mixtures were boiled at 100ºC for 10 min for protein denaturation. Equal masses of proteins from each sample were separated by electrophoresis on a 10% polyacrylamide SDS gel (Beyotime, Shanghai, China). The proteins were transferred onto 0.45 μm nitrocellulose membranes (Beyotime, Shanghai, China) and then blocked by 5% non-fatty milk for an hour. Primary antibodies were added to the membranes and incubated overnight. After washing 3 times (10 min per time) in PBST, (PBS with 0.1% Tween20), the blots were incubated with the secondary antibody (anti-mouse or anti-rabbit) for 60 min at room temperature. After washing with PBST, the membranes were detected by an Odyssey Scanning system (Li-Cor, USA) and measured by ImageStudio.

### FISH assay

The fluorescence *in situ* hybridization (FISH) probe of hsa_circ_0053063 was purchased from RiboBio (Guangzhou, China). The process of FISH followed the manufacturer’s instructions. 4', 6-Diamidino-2-Phenylindole (DAPI) was used for nuclear staining. The photographs were captured by the Thermo Fisher microscope (Thermo Fisher Scientific, Massachusetts, USA).

### Immunohistochemical assay

The immunohistochemical assay was performed on formalin-fixed, paraffin-embedded (FFPE) nude mouse tissues. FFPE slides were cut and prepared. Antibody PDCD4 was used to do IHC following the protocol. The slides of two groups were observed under the microscope, and five different views were photographed randomly per slide.

### Xenograft tumor assay

4–6 weeks old athymic female nude mice which weighed between 18 to 22 g were ordered from the laboratory animal center of Shanghai. Almost 1 × 10^6^ MDA-MB-231 transfected with lv-circ_0053063 or lv-NC were injected into the second mammary fat of each mouse (4 mice per group). Then, tumor size was measured and calculated every week. After 35 days, they were executed and their tumor lumps were collected immediately.

### Statistical analysis

The significant differences were assessed by GraphPad Prism V8.0 (GraphPad, CA, USA), SPSS 20.0 (IBM, SPSS, IL, USA), and RStudio (RStudio, MA, USA). Three independent experiment data were obtained and presented as the means ± standard deviation (SD). *P*-value < 0.05 was considered as significant results.

### Availability of data and materials

The datasets used and analyzed during the current study are available from the corresponding author on reasonable request.

### Ethics approval

Animal experiments were conducted in mice using protocols approved by the Ethics Committee of Shanghai Tenth People's Hospital of Tongji University, and written informed consent was obtained from all patients or their relatives.

## Supplementary Material

Supplementary Figure

Supplementary Table
