# Supplementary material for: Hsa_circ_0053063 inhibits breast cancer cell proliferation via hsa_circ_0053063/hsa-miR-330-3p/PDCD4 axis
Source: Aging (Albany NY). 2021 Mar 19;13(7):9627–45. doi: 10.18632/aging.202707 (PMC8064214; doi:10.18632/aging.202707)
Supplement: Supplementary Figure [file aging-13-202707-s001.pdf]

SUPPLEMENTARY FIGURE

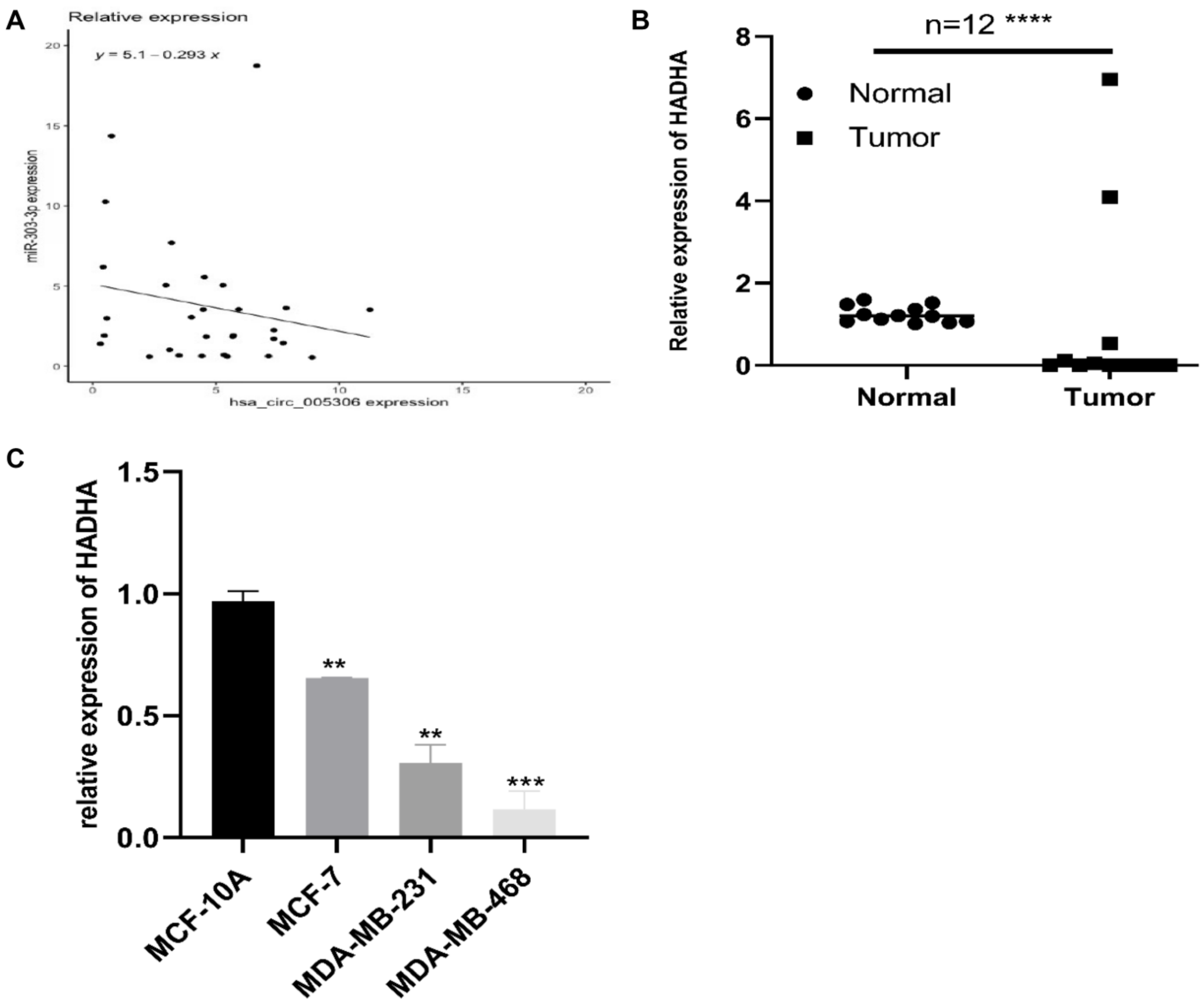

a

Supplementary Figure 1. Relationship between hsa_circ_0053063 and miR-330-3p and relative expression of HADHA in breast cancer tissues and breast cancer cell lines.
