# Supplementary material for: Hsa_circ_0053063 inhibits breast cancer cell proliferation via hsa_circ_0053063/hsa-miR-330-3p/PDCD4 axis
Source: Aging (Albany NY). 2021 Mar 19;13(7):9627–45. doi: 10.18632/aging.202707 (PMC8064214; doi:10.18632/aging.202707)
Supplement: Supplementary Table [file aging-13-202707-s002.pdf]

SUPPLEMENTARY TABLE

Supplementary Table 1. Primers and siRNAs used in this study.

PDCD4 siRNA	Sense	5'-GGAGCGGUUUGUAGAAGAAdTdT-3'
	Antisense	3'-dTdTCCUCGCCAAACAUCUUCUU-5'
PDCD4	Forward	5'-TCGTCGTTACGATTGGTTAGTC-3'
	Reverse	5'-GAAAAATCTCTAACCCTTCTCGC-3'
Has_circ_0053063	Forward	5'-CAAGACCCTTCAAGAAGTAACAC-3'
	Reverse	5'-AACCTCAAGTCCTCCTCCC-3'
GAPDH	Forward	5'-CAGGAGGCATTGCTGATGAT-3'
	Reverse	5'-GAAGGCTGGGGCTCATTT-3'
U6	Forward	5'-CAAATTCGTGAAGCGTTCCATAT-3'
	Reverse	5'-GCTTCACGAATTTGCGTGTCATCCTTGC-3'
18S rRNA	Forward	5'-GGACATCTAAGGGCATCACAG-3'
	Reverse	5'-GAGACTCTGGCATGCTAACTAG-3
